# Natural Cannabichromene (CBC) Shows Distinct Scalemicity Grades and Enantiomeric Dominance in *Cannabis sativa* Strains

**DOI:** 10.1021/acs.jnatprod.2c01139

**Published:** 2023-04-06

**Authors:** Andrea Calcaterra, Gabriele Cianfoni, Carola Tortora, Simone Manetto, Giulio Grassi, Bruno Botta, Francesco Gasparrini, Giulia Mazzoccanti, Giovanni Appendino

**Affiliations:** † Dipartimento di Chimica e Tecnologie del Farmaco, Sapienza Università di Roma, p.le A. Moro 5, 00185 Rome, Italy; ∧ Center for Life Nano- and Neuroscience at Sapienza, Italian Institute of Technology, Viale Regina Elena 291, 00161 Rome, Italy; § Canvasalus Research, Via Cristoforo Colombo 64, 35043 Monselice (PD), Italy; ‡ Dipartimento di Scienze del Farmaco, Largo Donegani 2, 28100 Novara, Italy

## Abstract

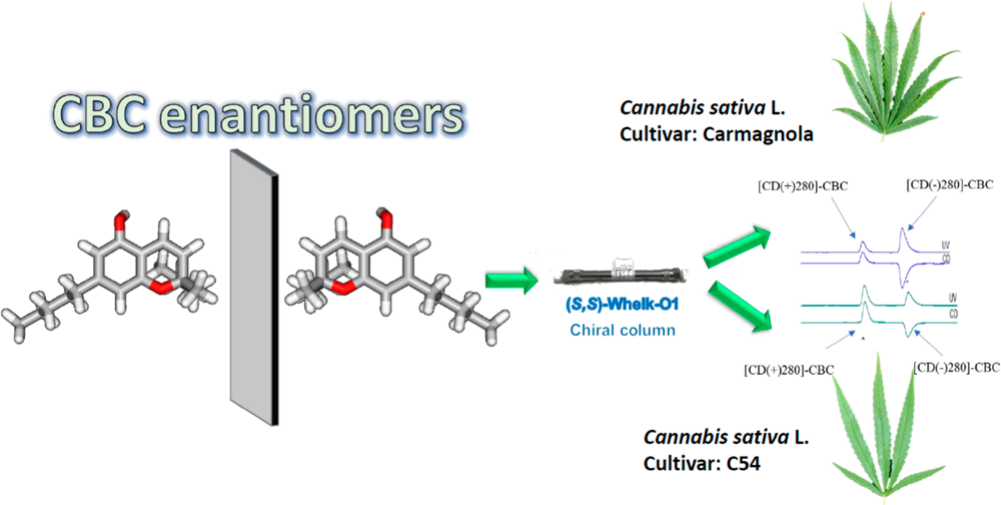

Cannabichromene (CBC, **1a**) occurs in Cannabis (*Cannabis sativa*) as a scalemate having a composition that is strain-dependent in terms of both enantiomeric excess and enantiomeric dominance. In the present work, the chirality of CBC (**1a**), a noncrystalline compound, was shown not to be significantly affected by standard conditions of isolation and purification, and enantiomeric self-disproportionation effects were minimized by carrying out the chiral analysis on crude fractions rather than on purified products. A genetic basis for the different enantiomeric state of CBC in Cannabis therefore seems to exist, implying that the chirality status of natural CBC (**1a**) in the plant is associated with the differential expression of CBCA-synthase isoforms and/or of associated directing proteins with antipodal enantiospecificity. The biological profile of both enantiomers of CBC should therefore be investigated independently to assess the contribution of this compound to the activity of Cannabis preparations.

The non-narcotic phytocannabinoid cannabichromene (CBC, **1a**) has a checkered history.^1^ It was long confused with cannabidiol (CBD, **2**) and considered a major constituent of marijuana (narcotic *Cannabis sativa* L.) on account of the isobaric relationship and similar chromatographic behavior of the two compounds.^2^ However, later studies clarified that CBC is a minor or even a trace constituent of *C*. *sativa* and its derived products (hashish, marijuana)^1^ and identified its major site of production and storage in sessile trichomes located mainly on the surface of young leaves and structurally distinct from the stalked trichomes where CBD and Δ^9^-tetrahydrocannabinol (Δ^9^-THC, **3**) are synthesized and accumulated.^3^

**Figure 1:**
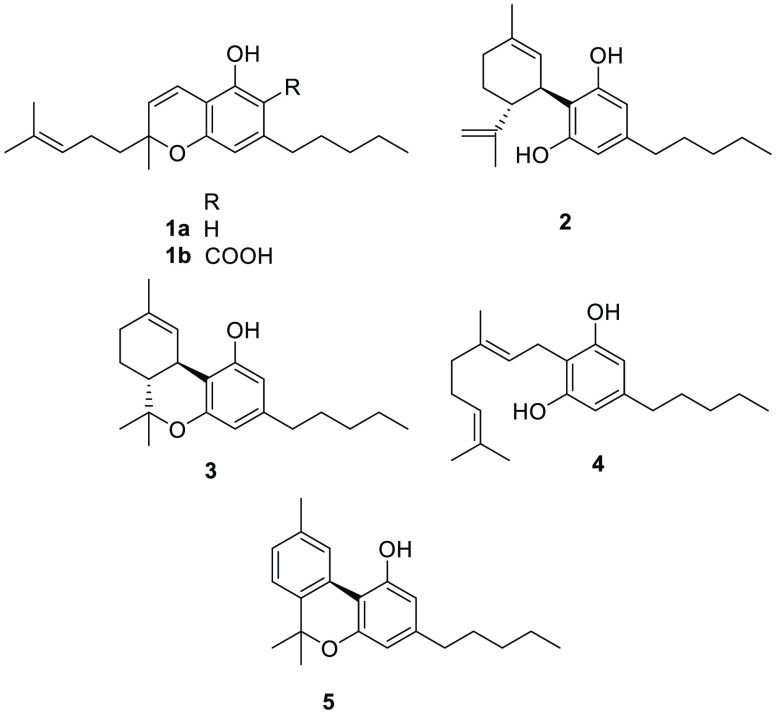
Structures of CBC (**1a**), CBCA (**1b**), CBD (**2**), Δ^9^-THC (**3**), CBG (**4**), and CBN (**5**).

Despite a difficult access from its natural source, CBC (**1a**) is easily available by synthesis from the tandem Knoevenagel–electrocyclization of citral and olivetol.^4^ This synthetic material was used in the bioactivity studies that led to the discovery that CBC is a potent and selective CB2^5^ and TRPA1^6^ agonist, providing a mechanistic basis for its powerful anti-inflammatory activity,^7^ while a remarkable antiepileptic activity was also reported.^8^ The observation that CBC has a better oral absorption compared to CBD (**2**) and Δ^9^-THC (**3**)^9^ provided an additional incentive for clinical studies, which are so far ongoing only with combination products.^9^

CBC (**1a**) is chiral and was originally isolated in 1966 in an optically active form,^10,11^ as was its acidic precursor (cannabichromenic acid, CBCA, **1b**).^12^ However, later studies concluded that CBC is racemic, with any residual optical activity and with positivity in the dog ataxia test being due presumably to the presence of impurities.^13^ By capitalizing on the inverted chirality column approach (ICCA) and enantioselective ultra-high-performance liquid chromatography (NP-eUHPLC), we reported that natural CBC is actually scalemic, with a prevalence of the [CD(+)280]-CBC.^14^ An independent study, based on chiral chromatography and deracemization of a crystalline derivative with (*S*)-ibuprofen for configurational assignment, confirmed the scalemic state of natural CBC but, surprisingly, reported a prevalence of the (*R*)-enantiomer (i.e., [CD(−)280]-CBC).^15^ Self-disproportionation of enantiomers (SDE) can, in principle, occur to some extent whenever a scalemate is subjected to phase partition like in crystallization or chromatography,^16^ and the optical purity of chromenes, generally good under laboratory conditions,^15^ can nevertheless be eroded by a light-induced equilibration with a quinone-methide form.^17^ This effect could, in principle, explain the different optical purity of CBC reported in different studies. Still, the different conclusions on the identity of the dominant enantiomer of natural CBC are surprising and prompted a systematic comparative study of the optical purity and enantiomeric dominance of CBC in different strains of *C*. *sativa*.

## Results and Discussion

**Figure 2:**
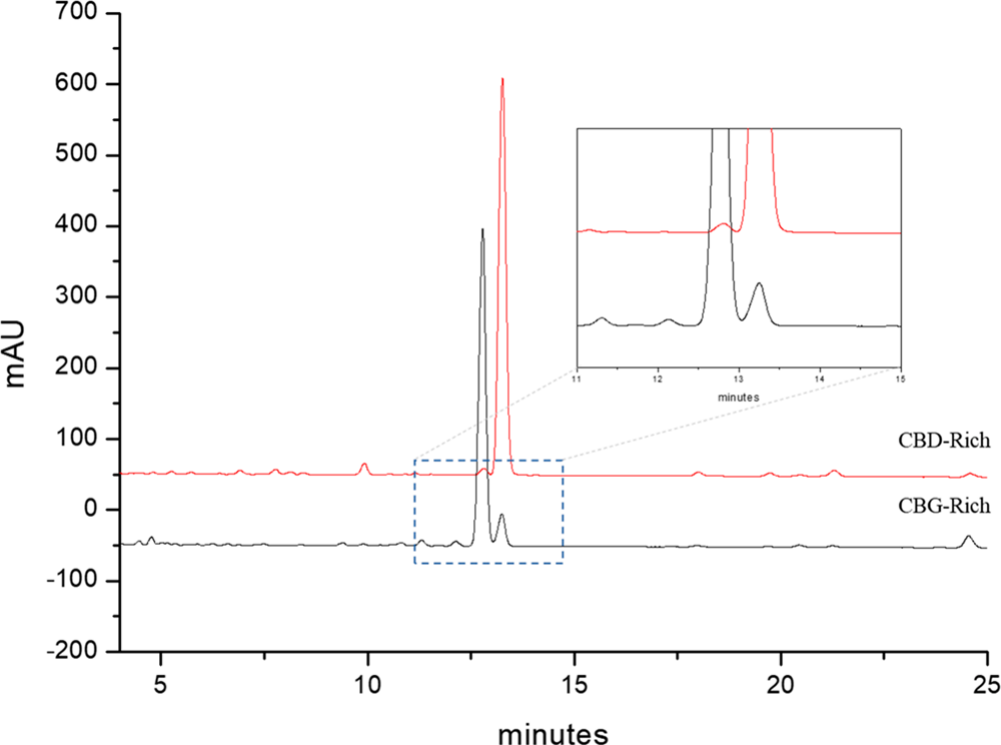
Crude plant ethanol extracts of CBG-rich and CBD-rich Cannabis strains (the red-line chromatogram refers to Finola, and the black-line chromatogram to the C37 cultivar).

Leaves from 10 different strains of fiber hemp were collected before anthesis or, in one case, also during and after anthesis. All samples were subjected to thermal decarboxylation of the biomass and ethanol extraction; then the crude ethanol extract was analyzed quantitatively by reversed-phase ultra-high-performance liquid chromatography (RP-UHPLC) (see Figure S1 in the Supporting Information for linearity, LOD, and LOQ values, and Figure S2 and Table S3 for chromatograms of standard mixture and resolutions values, respectively). The samples contained CBD (**2**) or CBG (**4**) as the dominant phytocannabinoid and low amounts of Δ^9^-*trans-*THC (**3**) (from below the detection threshold up to 0.16%). Figure 2 shows examples of chromatographic profiles of crude plant ethanol extracts of CBG-rich and CBD-rich Cannabis strains (C37 and Finola, respectively).

Major cannabinoids were quantified, and their concentrations as percentage (% w*/*w) values are reported in Table 1. In all samples, CBC (**1a**) was a relatively minor constituent, in the range of 0.02–0.67% of dry weight, with titers overall higher in the CBG varieties than in the CBD-rich strain.

**Table 1:** Cannabinoid Content (% w/w) in Cannabis Strains Characterized by Different Concentrations of CBD (**2**), CBG (**4**), CBN (**5**), Δ^9^-THC (**3**), and CBC (**1a**)^a^

	**entry**	**cultivar**	**(4)**	**(2)**	**(5)**	**(3)**	**(1a)**
CBD-rich	1	Carmagnola AZ Ventura	0.18	0.77	0.10	0.05	0.02
	2	Finola	N/A	6.42	0.07	0.05	0.14
	3	Antal	N/A	7.87	0.06	0.09	0.18
	4	Orange	0.21	8.87	0.05	0.16	0.43
	5	Carmagnola Az Green Lake	N/A	6.05	0.08	0.03	0.29
CBG-rich	6a	C37 (indoor)	4.12	0.27	0.01	N/A	0.51
	6b	C37 (outdoor)	4.15	1.83	N/A	0.02	0.51
	7a	C54 (indoor, leaves, preanthesis)	0.11	N/A	0.14	N/A	0.67
	7b	C54 (indoor, leaves, anthesis)	0.07	N/A	0.08	N/A	0.57
	7c	C54 (indoor, flowerheads, anthesis)	0.13	N/A	N/A	N/A	0.66
	7d	C54 (indoor, flower heads, postanthesis)	0.09	N/A	N/A	N/A	0.41
	7e	C54 (outdoor, leaves preanthesis)	0.72	N/A	N/A	N/A	0.53
	7f	C54 (outdoor, flowerheads)	0.06	N/A	N/A	N/A	0.32
	8	Gerona	0.22	N/A	N/A	N/A	0.07
	9	C53	0.06	N/A	N/A	N/A	0.63

a Data obtained by RP-UHPLC analysis and refer to leaves, unless specified otherwise.

**Figure 3:**
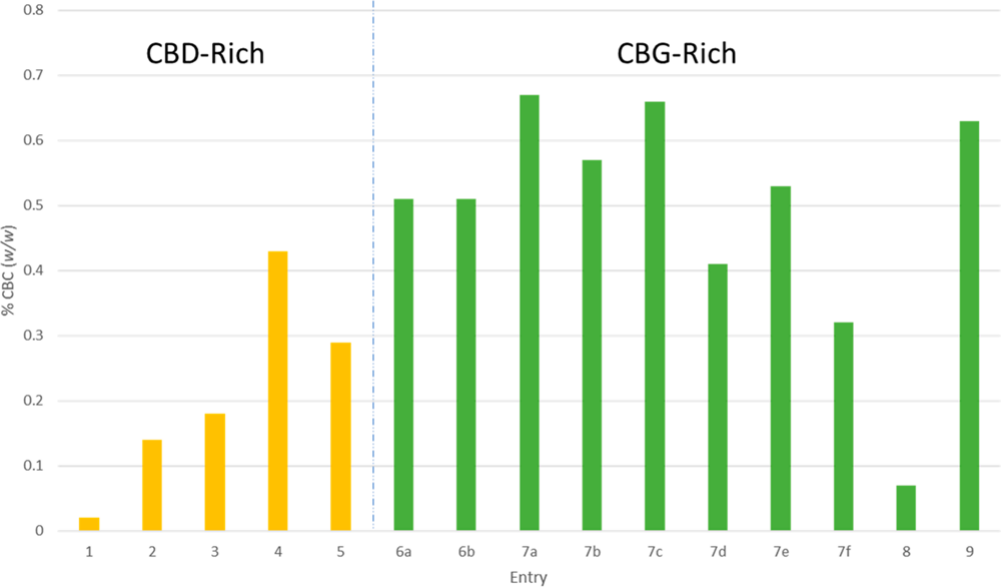
Bar-chart graph showing the CBC (**1a**) content (% w/w) for cultivars reported in Table 1.

Figure 3 reports the CBC content (% w/w) for the cultivars examined, and, in the single strain where leaves were collected at different development stages (i.e., entries 7a–f), the contents of CBC decreased during and after anthesis, in accordance with previous observations of higher CBC concentrations in juvenile tissues.^18^

After RP-UHPLC analysis, a crude CBC-enriched fraction was obtained by preparative TLC and analyzed by normal-phase enantioselective UHPLC using the ICCA strategy previously validated for the chiral analysis of phytocannabinoids.^14^ By calculation of the relative (%) area of the two enantiomers, the enantiomeric excess (ee) of CBC (**1a**) was calculated in all samples. Values ranging from 3% to 48% were observed, with the nature of the dominant enantiomer differing within the samples analyzed (Table 2).

**Table 2:** Relative Area (%) Values of the Two Enantiomers of CBC (**1a**) and Enantiomeric Excess (ee) for Cannabis Strains Obtained by Enantioselective NP-UHPLC Using Two (*R*,*R*)-Whelk-O1 and (*S*,*S*)-Whelk-O1 Columns^a^

			(*S*,*S*)-Whelk-O1			(*R*,*R*)-Whelk-O1		
	entry	cultivar	[CD(+)280]-CBC (rel.area %)	[CD(−)280]-CBC (rel.area %)	ee (%)	[CD(−)280]-CBC (rel.area %)	[CD(+)280]-CBC (rel.area %)	ee (%)
CBD-rich	1	Carmagnola AZ Ventura	52.96	47.04	5.92	46.22	53.78	7.56
	2	Finola	55.32	44.68	10.64	46.09	53.91	7.82
	3	Antal	55.50	44.5	11.00	43.69	56.31	12.62
	4	Orange	55.66	44.34	11.34	45.64	54.36	8.72
	5	Carmagnola Az Green Lake	34.06	65.94	31.88	67.98	32.02	35.96
CBG-rich	6a	C37 (indoor)	26.06	73.94	47.88	74.18	25.82	48.32
	6b	C37 (outdoor)	33.25	66.75	33.50	66.75	33.25	33.50
	7a	C-54 (indoor, leaves, preanthesis)	69.14	30.86	38.28	30.71	69.29	38.58
	7b	C-54 (indoor, flower heads, anthesis)	71.53	28.47	43.06	29.13	70.87	41.74
	7c	C-54 (indoor, flower heads, anthesis)	69.67	30.33	39.34	29.91	70.09	40.18
	7d	C-54 (indoor, flower heads, postanthesis)	62.31	37.69	24.62	38.76	61.24	22.48
	7e	C54 (outdoor, leaves preanthesis)	69.25	30.75	38.50	30.20	69.80	39.60
	7f	C54 (outdoor, flower heads)	68.60	31.40	37.20	33.36	66.64	33.28
	8	Gerona	70.48	29.52	40.96	32.36	67.64	35.28
	9	C53	51.99	48.01	3.98	48.48	51.52	3.04

a The strains where the dominant enantiomer is the opposite than detected in ref (14) are reported in gray.

**Figure 4:**
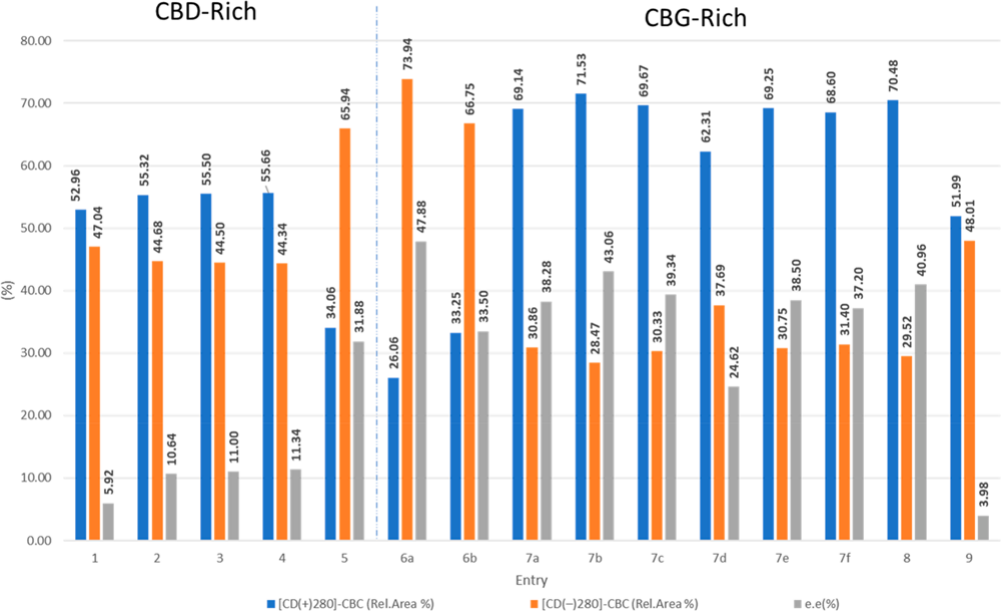
Relative percentage areas (%) of the CBC (**1a**) enantiomer peaks in the cultivars reported in Table 2 as resolved on an (*S*,*S*)-Whelk-O1 column (first eluted in blue, second eluted in orange). The enantiomeric excesses (ee %) are reported as gray bars.

The ICCA approach made it possible to avoid the coelution of impurities with retention times similar to those of the enantiomers under relative quantification, resulting in a more precise and reliable quantification of the ee, despite small variations between the values recorded from one column to another. In this work, the two enantiomers of CBC (**1a**) were discriminated based on the sign they showed on the ECD trace set at 280 nm (Figure S4 in the Supporting Information). No relationship between the dominance of a specific phytocannabinoid (CBD or CBG) and that of a specific enantiomer of CBC was observed, and no significant variation of the ee was observed during plant development (see Figure 4).

**Scheme 1:**
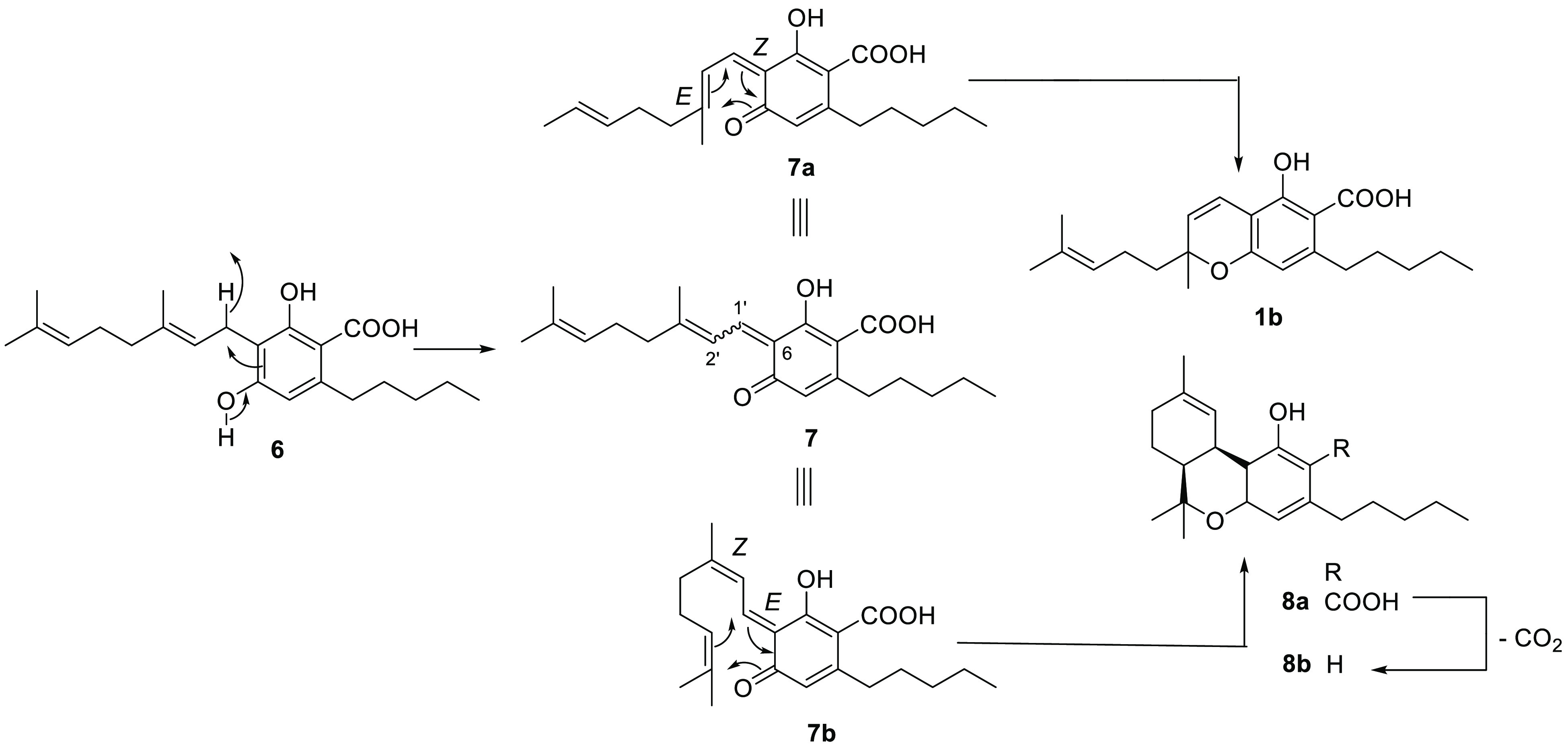
Formation of CBCA (**1b**) and *cis*-Δ^9^-THCA (**8a**) from the Electrocyclization of the Diastereomeric Forms of the Quinone Methide Intermediates **7a** and **7b**

The optical purities of chiral chromenes are scarcely affected by heat.^17^ Still, they are significantly eroded by UV exposure,^17^ and there is the possibility that racemization could, to a certain extent, potentially affect the enantiomeric composition of CBC (**1a**) when data from different laboratories are compared. Nevertheless, the discovery of opposite enantiomeric dominance in different strains strongly suggests an enzymatic origin for the variation of the scalemate composition. It is not uncommon that the composition of natural scalemates significantly differs in related plants or even in different organs of the same plant.^19^ For mono- and sesquiterpenoids, this has been associated with the different expression of synthases with complementary enantioselectivity,^20,21^ while the chirality status of scalemic lignans has been shown to depend on the expression of enantiomeric directing proteins.^19^ CBCA (**1b**), the native form of CBC (**1a**), is derived from CBGA (**4**) by the agency of a specific oxidase (CBCA-synthase), a member of the berberine bridge enzyme (BBE)-like gene family.^22−24^ The enzyme oxidizes the resorcinol core of CBGA (**4**) to an achiral quinone methide (**5a**), which next spontaneously electrocyclizes to CBCA (**1b**) (Scheme 1). Early studies on a partially purified enzyme suggested the formation of a 5:1 scalemate of CBCA,^22^ but additional confirmation was not reported, and nor was the dominant enantiomer identified or the presence of the directing proteins investigated. Oxidation to a quinone methide is also involved in the dimerization of phenylpropane alcohols (monolignols) to lignans, where an oxidative enzymatic phase is followed by a nonenzymatic coupling assisted by enantiospecific directing proteins.^19^ The enantiomeric status of CBC therefore could result from the involvement of synthases or of directing proteins with antipodal enantioselectivity. Further studies will be necessary to clarify this issue, which is of general relevance for the whole class of chromene and chromene-derived scalemates.^25^ Remarkably, a similar tandem oxidation–electrocyclization of CBGA (**6**) might be involved in the scalemic state of *cis*-THC (**8b**),^26^ of which the acidic precursor (**8a**) could derive from the *E*-Δ^1′,6^ isomer **7b** of the quinone methide involved in the biogenesis of CBCA (**1b**) (Scheme 1).

## Conclusions

Cannabichromene (**1a**) occurs in Cannabis (*Cannabis sativa* L.) as a scalemate having a composition that is strain-dependent in terms of both enantiomeric excess and enantiomeric dominance. Due to the discovery of the potent synergistic activity of CBC (**1a**) with Δ^9^-THC and possibly other cannabinoids as well,^27^ there is a growing interest in the generation of Cannabis strains that retain a juvenile metabolic trait and accumulate substantial amounts of CBC (**1a**) in addition to other cannabinoids.^8,17^ On the other hand, the bioactivity profiles of enantiomers rarely overlap,^28^ as could the biological profile of scalemic and natural CBC (**1a**), highlighting the need to independently investigate the biological profile of both enantiomers for a proper evaluation of the role of natural scalemic CBC in Cannabis preparations. Due to the powerful anticonvulsant activity of (racemic) synthetic CBC,^7^ this seems especially urgent for studies aimed at the management of intractable pediatric epilepsy.

## Experimental Section

### General Experimental Procedures

All solvents used for UHPLC analyses were HPLC grade and were purchased from Merck Life Science (Milan, Italy) and Carlo Erba Reagents (Milan, Italy). Cannabinoid reference standards dissolved in methanol, namely, cannabidivarin (CBDV, 1 mg/mL), cannabidiolic acid (CBDA, 1 mg/mL), cannabigerol (CBG, 1 mg/mL), cannabidiol (CBD, 1 mg/mL), cannabinol (CBN, 1 mg/mL), (−)-Δ^9^-tetrahydrocannabinol [(−)-Δ^9^-THC, 1 mg/mL], (−)-Δ^8^-tetrahydrocannabinol [(−)-Δ^8^-THC, 1 mg/mL], cannabichromene (CBC, 1 mg/mL), and (−)-Δ^9^-tetrahydrocannabinolic acid ((−)-Δ^9^-THCA, 1 mg/mL) with a purity of ≥99%, were purchased from Merck Life Science (Milan, Italy). Preparative TLC plates UV 254 nm (surface 20 × 20 cm, thickness 500 μm), used for the purification of CBC, were purchased from Merck (Darmstadt, Germany).

### Plant Material

All Cannabis samples were provided by Canvasalus Research, Via Cristoforo Colombo 64, 35043 Monselice (PD), Italy, and were collected during the 2021–2022 growing seasons.

### Ultrasonic-Assisted Extraction

The dried plant material (100 mg) was decarboxylated by heating to 130 °C for 2 h in a glass test tube. The plant material was then extracted with analytical-grade ethanol (5 mL) in an ultrasound bath for 30 min. The extract was filtered through a 0.45 μm PTFE membrane and then analyzed by UHPLC.

### Cannabinoid Content by RP-UHPLC Analyses

Analyses were carried out by using an Ultimate 3000 ultra-high-performance liquid chromatograph (Thermo Fisher Scientific, Waltham, MA, USA), with a binary gradient system, an automatic injector, a thermostatic column compartment, and a diode array detector. The system was controlled by Chromeleon 7.2 Chromatography Data System software (Thermo Fisher Scientific, 1.0.5. v). All separations were performed by using a Titan C_18_ column (100 × 3 mm, l × i.d., 1.9 μm) (Sigma-Aldrich, St. Louis, MO, USA) with a mobile phase composed of 0.1% formic acid in both (A) water and (B) acetonitrile. The total run time was 22 min, and the chromatographic conditions were set as follows: 65% B (0 min), 100% B (14 min), 100% B (16 min), 65% B (17 min), and 65% B (22 min). The flow rate was 0.5 mL/min. The column temperature was set at 35 °C. A volume of 5 μL was injected. The PDA detector was set to 214 nm wavelength. To prepare the calibration curves (Supporting Information, Figures S1–S9), standard cannabinoid solutions of CBDV, CBD, CBC, CBDA, CBG, CBN, Δ^8^-THC, Δ^9^-THC, and Δ^9^-THCA were prepared in the concentration range from 0.001 to 0.05 mg/mL. Regression lines were calculated using the least-squares method, and linearity was expressed by the determination coefficient (*R*^2^). For each calibration curve, the *R*^2^ value was always greater than 0.997, showing good linearity. The limit of detection (LOD) was in the range of 0.002–0.003 mg/mL, and the limit of quantitation (LOQ) was in the range of 0.007–0.009 mg/mL. Cannabinoid concentrations were expressed as percentages (w/w).

### Isolation of a CBC (**1a**)-Enriched Fraction by Preparative TLC

The crude extract of each cultivar dissolved in MeOH (HPLC grade) was carefully deposited on a preparative TLC plate (20 × 20 cm). The preparative TLC chamber was provided with 150 mL of eluent, consisting of hexane/EtOAc (9:1) + 1.5 mL of CHCl_3_. The preparative TLC plate was placed inside the chamber and run for 1 h. After this time, the plate was removed and the band corresponding to CBC (*R_f_* = 0.34) was visualized and identified under UV light (254 nm). This band was then scratched from the plate, and the silica gel was placed in a 20 mL vial, diluted with 25 mL of MeOH (HPLC grade), and extracted in an ultrasonic bath for 15 min. The mixture was filtered using a filter paper to remove the silica, and the solvent was removed under reduced pressure from the methanol extract. UHPLC samples were prepared by diluting the residue in the mobile phase.

### Enantioselective NP-eUHPLC Chromatographic Analysis and ICCA Application

Enantioselective UHPLC analyses of CBC, isolated as described above, were performed on an UltiMate 3000RSLC (Dionex, Benelux, Amsterdam, The Netherlands). Specifically, the instrument operation and chromatographic data acquisition and processing were performed using the Chromeleon 7.2 chromatography data system. All separations were performed using (*R*,*R*)-Whelk-O1 and (*S*,*S*)-Whelk-O1 CSPs, prepared according to a previously described procedure starting from Kromasil 1.8 μm silica particles and slurry packed into 100 × 4.6 mm (L × i.d.) stainless steel columns and commercially available from Regis Technologies Inc. (Morton Grove, IL, USA). Isocratic conditions were set as follows: mobile phase: *n*-hexane/isopropanol (99.5:0.5 v*/*v); flow rate: 1.0 mL/min; *T* = 30 °C; detection: UV 280 nm, CD 280 nm.

## Supporting Information Available

Calibration curves, RP-UHPLC chromatograms, and chromatograms for the determination of enantiomeric excesses (PDF)

## References

[ref1] Pollastro F.; Caprioglio D.; Del Prete D.; Rogati F.; Minassi A.; Taglialatela-Scafati O.; Muñoz E.; Appendino G. Nat. Prod. Commun. 2018, 13, 1189–1194. 10.1177/1934578X1801300922.

[ref2] Turner C. E.; Hadley K. W.; Holley J. H.; Billets S.; Mole M. L. Jr. J. Pharm. Sci. 1975, 64, 810–814. 10.1002/jps.2600640517. 1151651

[ref3] Mahlberg P. G.; Kim E. S. J. Ind. Hemp 2004, 9, 15–36. 10.1300/J237v09n01_04.

[ref4] ElSohly H. N.; Turner C. E.; Clark A. M.; ElSohly M. A. J. Pharm. Sci. 1982, 71, 1319–1323. 10.1002/jps.2600711204. 7153877

[ref5] Udoh M.; Santiago M.; Devenish S.; McGregor I. S.; Connor M. Br. J. Pharmacol. 2019, 176, 4537–4547. 10.1111/bph.14815. 31368508 PMC6932936

[ref6] De Petrocellis L.; Ligresti A.; Schiano Moriello A.; Allarà M.; Bisogno T.; Petrosino S.; Stott C. G.; Di Marzo V. Br. J. Pharmacol. 2011, 163, 1479–1494. 10.1111/j.1476-5381.2010.01166.x. 21175579 PMC3165957

[ref7] Izzo A. A.; Capasso R.; Aviello G.; Borrelli F.; Romano B.; Piscitelli F.; Gallo L.; Capasso F.; Orlando P.; Di Marzo V. Br. J. Pharmacol. 2012, 166, 1444–1460. 10.1111/j.1476-5381.2012.01879.x. 22300105 PMC3417459

[ref8] Anderson L. A.; Ametovski A.; Luo J. L.; Everett-Morgan D.; McGregor I. S.; Banister S. D.; Arnold J. C. ACS Chem. Neurosci. 2021, 12, 330–339. 10.1021/acschemneuro.0c00677. 33395525

[ref9] Peters E. N.; MacNair L.; Mosesova I.; Christians U.; Sempio C.; Klawitter J.; Land M. H.; Ware M. A.; Turcotte C.; Bonn-Miller M. O. Eur. J. Clin. Pharmacol. 2022, 78, 259–265. 10.1007/s00228-021-03232-8. 34664109 PMC8748343

[ref10] Gaoni Y.; Mechoulam R. Chem. Commun. 1966, 20–21. 10.1039/c19660000020.

[ref11] Claussen U.; Spulak F.; Korte F. Tetrahedron 1966, 22, 1477–1479. 10.1016/S0040-4020(01)99445-1.

[ref12] Shoyama Y.; Fujita T.; Yamauchi T.; Nishioka I. Chem. Pharm. Bull. 1968, 16, 1157 10.1248/cpb.16.1157. 5706836

[ref13] Mechoulam R.; Yagnitinsky B.; Gaoni Y. J. Am. Chem. Soc. 1968, 90, 2418–2420. 10.1021/ja01011a037. 5642071

[ref14] Mazzoccanti G.; Ismail O. H.; D’Acquarica I.; Villani C.; Manzo C.; Wilcox M.; Cavazzini A.; Gasparrini F. Chem. Commun. 2017, 53, 12262–12265. 10.1039/C7CC06999E. 29072720

[ref15] Agua A. R.; Barr P. J.; Marlowe C. K.; Pirrung M. C. J. Org. Chem. 2011, 86, 8036–8040. 10.1021/acs.joc.1c00451. 34078070

[ref16] Han J.; Kitagawa O.; Wzorek A.; Klika K. D.; Soloshonok V. A. Chem. Sci. 2018, 9, 1718–1739. 10.1039/C7SC05138G. 29675218 PMC5892310

[ref17] Jeong H.; Kang G.; Kim M. J.; Shin J. S.; Han S.; Lee H.-Y. Org. Lett. 2022, 24, 2181–2185. 10.1021/acs.orglett.2c00482. 35266724

[ref18] de Meijer E. P. M.; Hammond K. M.; Micheler M. Euphytica 2009, 165, 293–311. 10.1007/s10681-008-9787-1.

[ref19] Davin L. B.; Lewis N. G. Plant Physiol. 2000, 123, 453–461. 10.1104/pp.123.2.453. 10859176 PMC1539258

[ref20] Phillips M. A.; Wildung M. R.; Williams D. C.; Hyatt D. C.; Croteau R. Archiv. Biochem. Biophys. 2003, 411, 267–276. 10.1016/S0003-9861(02)00746-4. 12623076

[ref21] Schmidt C. O.; Bouwmeester H. J.; Franke S.; König W. Chirality 1999, 11, 353–362. 10.1002/(SICI)1520-636X(1999)11:5/6<353::AID-CHIR2>3.0.CO;2-L.

[ref22] Shoyama Y.; Fujita T.; Yamauchi T.; Nishioka I. Chem. Pharm. Bull. 1968, 16, 1157 10.1248/cpb.16.1157. 5706836

[ref23] Morimoto S.; Komatsu K.; Taura F.; Shoyama Y. J. Nat. Prod. 1997, 60, 854–857. 10.1021/np970210y.

[ref24] Morimoto S.; Koimatsu K.; Taura F.; Shoyama Y. Phytochemistry 1998, 49, 1525–1529. 10.1016/S0031-9422(98)00278-7. 9862135

[ref25] Huang G.-H.; Hu Z.; Lei C.; Wang P.-P.; Yang J.; Jing-Ya Li J.-Y. Li; Hou A.-J. J. Nat. Prod. 2018, 81, 1810–1818. 10.1021/acs.jnatprod.8b00273. 30067363

[ref26] Schafroth M. A.; Mazzoccanti G.; Reynoso-Moreno I.; Erni R.; Pollastro F.; Caprioglio D.; Botta B.; Allegrone G.; Grassi G.; Chicca A.; Gasparrini F.; Gertsch J.; Carreira E. M.; Appendino G. J. Nat. Prod. 2021, 84, 2502–2510. 10.1021/acs.jnatprod.1c00513. 34304557

[ref27] DeLong G. T.; Wolf C. E.; Poklis A.; Lichtman A. H. Drug Alcohol Depend. 2010, 112, 126–133. 10.1016/j.drugalcdep.2010.05.019. 20619971 PMC2967639

[ref28] Mori K. Bioorg. Med. Chem. 2007, 15, 7505–7523. 10.1016/j.bmc.2007.08.040. 17855097

