# Supplementary material for: Natural Cannabichromene (CBC) Shows Distinct Scalemicity Grades and Enantiomeric Dominance in Cannabis sativa Strains
Source: J Nat Prod. 2023 Apr 6;86(4):909–14. doi: 10.1021/acs.jnatprod.2c01139 (PMC10152484; doi:10.1021/acs.jnatprod.2c01139)
Supplement: Supplementary file 1 — np2c01139_si_001.pdf [file np2c01139_si_001.pdf]

Supporting Information

Natural Cannabichromene (CBC) Shows Distinct Scalemicity Grades and Enantiomeric Dominance in *Cannabis sativa* Strains

Calibration curves **S1**

Chromatogram of cannabinoids standard mixture (RP-UHPLC) **S2**

Chromatographic resolutions RP-UHPLC **S3**

Chromatograms (UV and CD traces) NP-eHPLC of CBC isolated fractions **S4**

Calibration curves S1

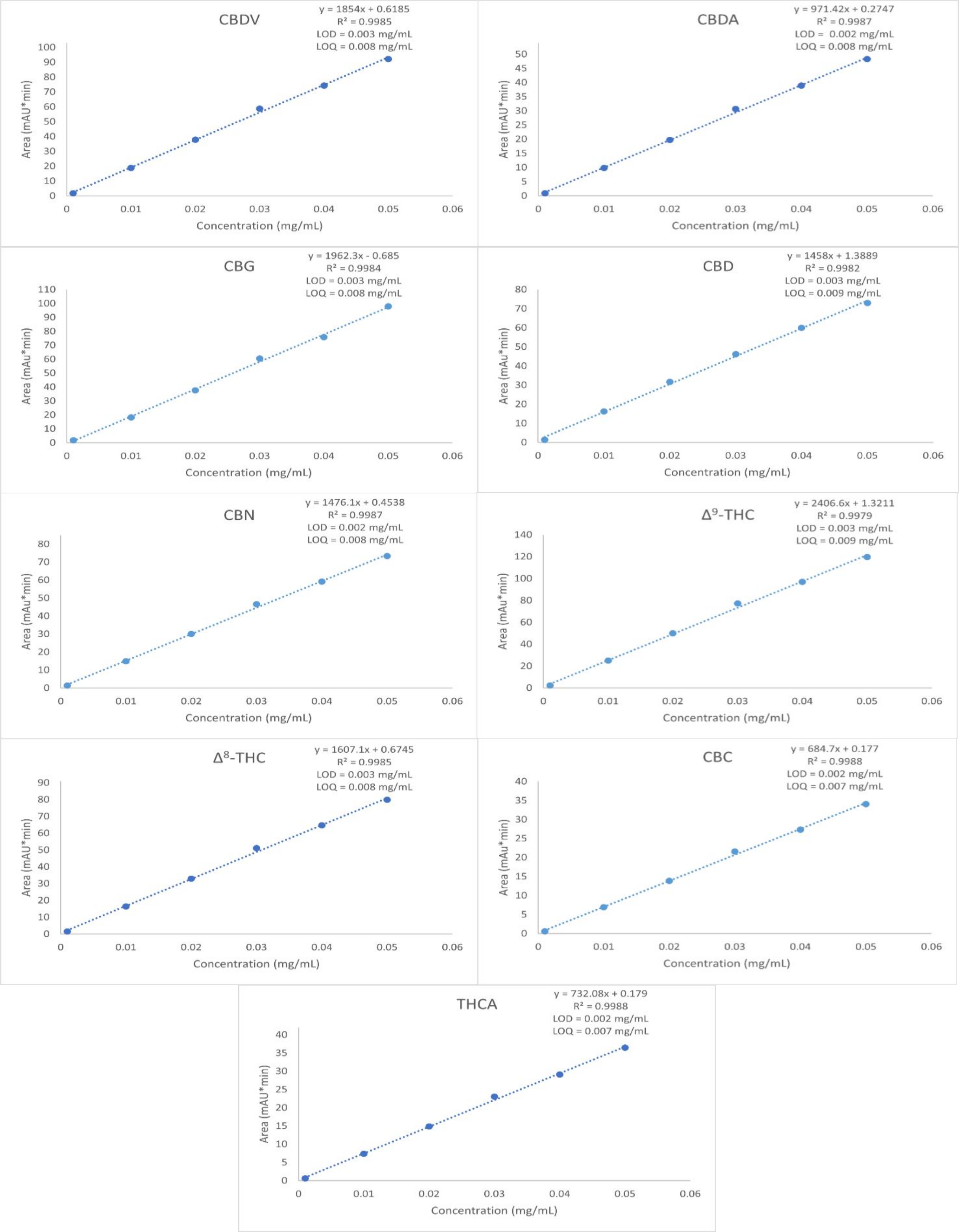

Figure S1. Calibration curves of standard cannabinoids.

Chromatogram of cannabinoids standard mixture (RP-UHPLC)

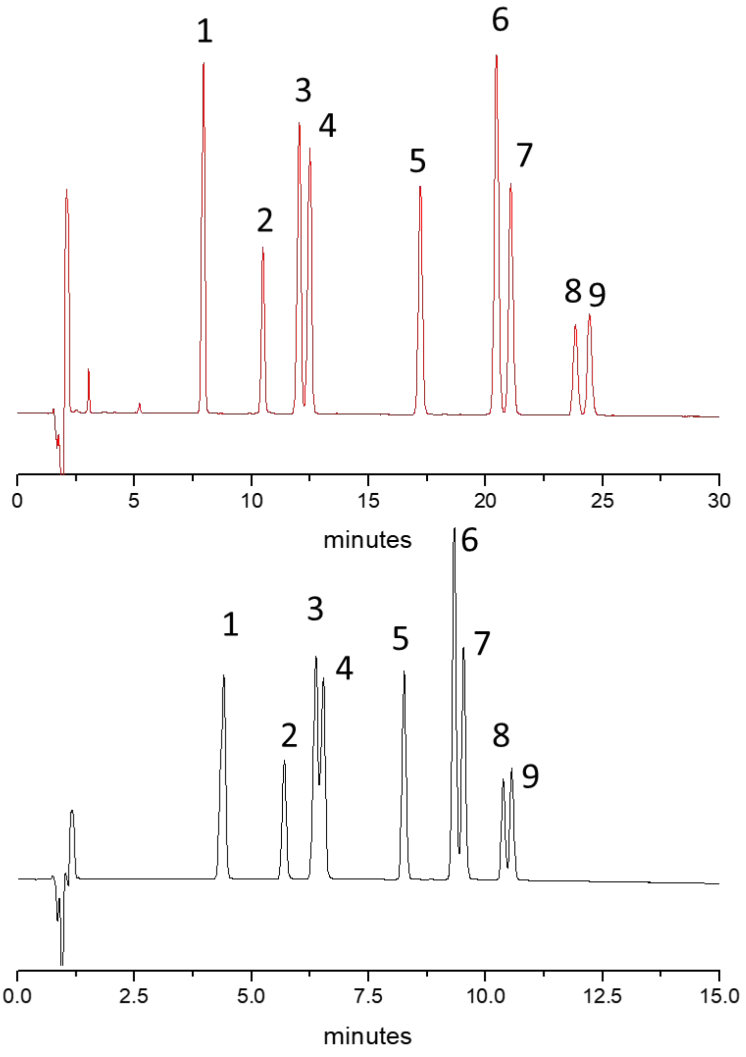

Figure S2. Chromatograms of standard cannabinoids mixture: CBDV (1), CBDA (2), CBG (3), CBD (4), CBN (5), Δ^9-THC (6), Δ^8-THC (7), CBC (8), THCA (9). Top) Elution conditions: two Titan C18 columns in series, flow rate 0.5 mL/min; Down) Elution conditions: one Titan C18 column, flow rate 0.5 mL/min.

Chromatographic resolutions RP-UHPLC

Table S1. Resolution registered for each cannabinoid in the two chromatographic separation conditions.

		1	2	3	4	5	6	7	8	9
one Titan C18 column	Resolution	n.a.	6.82	3.72	0.79	9.37	6.73	1.28	5.57	1.21
two Titan C18 columns in series	Resolution	n.a.	10.08	5.69	1.48	15.09	9.92	1.80	7.92	1.69

Chromatograms (UV and CD traces) NP-eHPLC of CBC isolated fractions

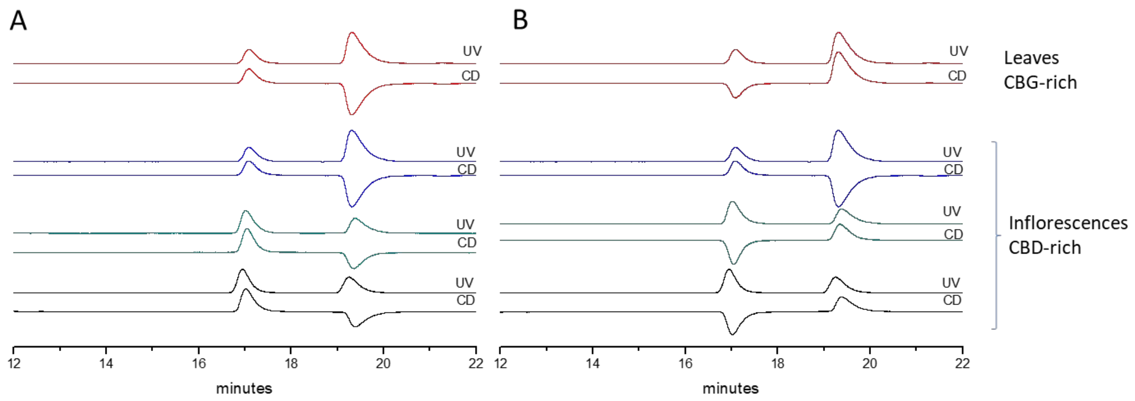

Figure S3. Chromatograms on (*S,S*)-WhelkO1 (A) and (*R,R*)-WhelkO1 (B) column of CBC purified fraction from Finola (black-traces), Orange (green-traces), Carmagnola AZ Greenlake (Blue-traces) and OGM1 C37 (red-traces) cultivars. Detection UV and CD at 280 nm.
